# Evaluation of the Diagnostic Performance of Prenatal Screening Markers for Down Syndrome in the Turkistan Region

**DOI:** 10.3390/healthcare14182943

**Published:** 2026-09-10

**Authors:** Zhansaya Torgauytova, Ardak Ayazbekov, Gulzhakhan Omarova, Almagul Kurmanova, Damilya Salimbayeva, Altynay Nurmakova, Natalya Kravtsova, Makhambet Smailov, Rinaliya Usmanova

**Affiliations:** 1 Faculty of Medical, Khoja Akhmet Yassawi University, 161200 Turkistan, Kazakhstan; jans.86@mail.ru; 2 Scientific Center for Obstetrics, Gynecology and Perinatology, 125 Dostyk Ave., 050010 Almaty, Kazakhstan; ardakayazbekov966@gmail.com (A.A.); sdamilya@mail.ru (D.S.); nat_kravtsova@mail.ru (N.K.); www.maha.uk@mail.ru (M.S.); rinaliya.usmanova@gmail.com (R.U.); 3 Faculty of Medicine and Healthcare, Farabi University, 71, Al-Farabi Avenue, 050040 Almaty, Kazakhstan

**Keywords:** Down syndrome, prenatal screening, nuchal translucency, pregnancy-associated plasma protein A, free beta-human chorionic gonadotropin

## Abstract

**Highlights:**

**What are the main findings?**

- Maternal age and fetal nuchal translucency (NT) showed the strongest associations with Down syndrome during first-trimester prenatal screening, whereas PAPP-A and free β-hCG did not retain independent statistical significance after multivariable adjustment.
- Incorporating nasal bone hypoplasia/aplasia into the prediction model improved the observed diagnostic performance, increasing the AUC from 0.855 to 0.903 while maintaining high specificity and demonstrating good calibration and internal validation.

**What are the implications of the main findings?**

- The findings may inform further evaluation of prenatal risk assessment strategies in high-birth-rate regions, while external validation is required before potential clinical application.
- The findings provide evidence for optimizing prenatal screening services in high-birth-rate regions and support the future integration of region-specific prediction models into clinical decision-support systems and personalized prenatal care programs following external validation.

**Abstract:**

**Background:** The Turkestan region is one of the regions with a high birth rate; therefore, analysis of the effectiveness of an early detection system for chromosomal abnormalities in this region is important for practical healthcare. **Objective:** To evaluate the diagnostic potential of first-trimester prenatal screening markers for identifying Down syndrome in the Turkestan region. **Methods:** A retrospective analytical study was conducted based on the analysis of medical records of pregnancies registered at perinatal centers in the Turkestan region between 2020 and 2025. The final analytical sample included 143 cases, comprising 71 pregnancies affected by Down syndrome and 72 randomly selected pregnancy cases included for comparative analysis. Maternal age, nuchal translucency (NT) thickness, pregnancy-associated plasma protein A (PAPP-A), free beta-human chorionic gonadotropin (free β-hCG), and fetal nasal bone hypoplasia/aplasia were assessed. Multivariable logistic regression and receiver operating characteristic (ROC) curve analysis were used. **Results:** In the multivariable analysis, maternal age (odds ratio [OR] = 1.16; *p* < 0.001) and NT (OR = 3.01; *p* = 0.001) showed statistically significant independent associations with Down syndrome. PAPP-A and free β-hCG did not demonstrate statistically significant independent associations in the multivariable model. The area under the ROC curve (AUC) was 0.855 for Model 1 and 0.903 for Model 2, which additionally included fetal nasal bone status. **Conclusions:** The exploratory models demonstrated good discriminatory performance within the present retrospective analytical sample. Maternal age and ultrasound markers showed the strongest associations with Down syndrome in the multivariable analysis. These models are not intended for direct clinical application at this stage and require external validation in independent and representative prenatal screening populations before their potential clinical utility can be assessed.

## 1. Introduction

Congenital malformations and chromosomal disorders are among the most important problems in modern perinatology and medical genetics. One of the most common chromosomal pathologies in this context is Down syndrome [1,2]. This condition is characterized by delayed intellectual development in children and malformations of many organs and systems, and imposes a significant medical and socioeconomic burden on society [2,3]. Currently, the development of prenatal diagnostic methods makes it possible to detect chromosomal abnormalities in the fetus at an early stage. The screening of pregnant women includes a comprehensive approach based on the assessment of biochemical markers (PAPP-A, free β-hCG) and ultrasound parameters (nuchal fold thickness, hypoplasia or aplasia of the nasal bone) [4]. These methods make it possible to identify pregnant women at risk and carry out invasive diagnostic procedures in a timely manner [5]. Despite the widespread implementation of national screening programs, the incidence of congenital malformations is approximately 2–3% of all pregnancies worldwide [6]. Moreover, in many cases, double or systemic congenital malformations are not the result of random morphological changes, but their pathogenetic basis is chromosomal abnormalities. According to the literature, 15–30% of such defects are associated with quantitative anomalies of the chromosomal apparatus [7]. However, a number of studies have shown that biochemical screening has limited informativeness in detecting Down syndrome, while ultrasound markers have high diagnostic value. Furthermore, the combined use of various screening markers has been shown to significantly improve diagnostic efficacy [8].

Taking into account regional characteristics, assessing the effectiveness of prenatal screening is of particular importance [9]. The Turkestan region is one of the regions with a high birth rate; therefore, analysis of the effectiveness of the early detection system for chromosomal pathologies in this region is important for practical healthcare [10].

Despite substantial advances in prenatal screening, important challenges remain in optimizing risk assessment strategies across different populations. The diagnostic performance of first-trimester screening algorithms may vary according to demographic characteristics, ethnic composition, and regional differences in healthcare delivery. Most widely used prediction models have been developed and validated in high-income countries, whereas evidence from Central Asian populations remains limited. Consequently, the applicability of these models to regions with distinct population structures and high birth rates has not been sufficiently investigated.

The Turkestan region represents one of the fastest-growing populations in Kazakhstan, with a high annual birth rate and increasing demand for high-quality prenatal screening services. Early identification of pregnancies at increased risk of Down syndrome is particularly important in this setting, as it enables timely genetic counseling, appropriate referral for confirmatory testing, and informed clinical decision-making. Improving the accuracy of first-trimester risk assessment may also reduce unnecessary invasive procedures while ensuring that high-risk pregnancies receive appropriate diagnostic evaluation.

Furthermore, the clinical effectiveness of prenatal screening and genetic diagnosis is determined not only by their diagnostic accuracy. Decisions regarding the uptake of prenatal genetic testing, acceptance of invasive diagnostic procedures, and the continuation of pregnancy following the detection of a fetal chromosomal abnormality may also be influenced by social, cultural, and religious factors. Studies conducted among different religious and cultural communities have demonstrated substantial differences in attitudes toward prenatal testing and pregnancy termination. These factors may influence women’s decisions to undergo prenatal diagnostic procedures, accept invasive testing, and make subsequent decisions based on the results obtained. Therefore, when evaluating and improving prenatal screening programs, it is important to consider not only their diagnostic characteristics but also the sociocultural and religious context of the population in which screening is implemented [11,12,13].

Recent studies have demonstrated the potential of multivariable models integrating first-trimester ultrasound and clinical markers to improve individualized risk assessment for trisomy 21, highlighting the importance of evaluating both discrimination and calibration when developing predictive models [14].

Although prenatal screening for fetal chromosomal abnormalities has been extensively studied internationally, evidence from Kazakhstan and Central Asia remains limited. Available studies from Kazakhstan have primarily focused on biochemical screening outcomes or the overall epidemiology of congenital anomalies. In one Kazakhstani study, 30,335 pregnant women underwent first- and second-trimester biochemical screening; 662 women were classified as being at high risk, and invasive prenatal diagnosis identified fetal abnormalities in 41 cases, including 29 cases of Down syndrome [15]. In the Turkestan region, 848 registered cases of congenital anomalies were reported among 53,169 pregnancies between 2020 and 2024, with the registered prevalence increasing from 0.8% to 2.4% during this period [15]. However, these studies did not evaluate the diagnostic and predictive performance of combined first-trimester maternal, biochemical, and ultrasound markers specifically for Down syndrome. Therefore, evidence regarding the evaluation of multivariable models based on combined first-trimester screening parameters in the local population remains insufficient.

Therefore, the present study aimed to evaluate the diagnostic performance of established first-trimester screening markers for Down syndrome and to explore their combined performance in multivariable models in a regional population with a high birth rate. The findings may provide evidence to inform further research on prenatal screening strategies in Kazakhstan and other settings with similar demographic characteristics.

We hypothesized that a multivariable model incorporating maternal age, first-trimester ultrasound markers, and biochemical screening markers would demonstrate good discriminatory performance for identifying pregnancies affected by Down syndrome. We further hypothesized that the inclusion of fetal nasal bone hypoplasia/aplasia would improve the discriminatory performance of the model.

## 2. Materials and Methods

### 2.1. Study Design and Participants

This retrospective analytical study was conducted in the Turkestan region, Republic of Kazakhstan, and was based on the analysis of medical records from regional and district perinatal centers between 2020 and 2025. All cases of Down syndrome registered at these centers during the study period were included in the initial study population.

A total of 103 cases of Down syndrome were registered during the study period. Of these, 80 were live-born children with Down syndrome, while in 23 cases, fetal Down syndrome was diagnosed prenatally and confirmed by invasive testing using amniocentesis, after which the pregnancies were terminated. Among the 80 live-born cases, the diagnosis was confirmed prenatally by amniocentesis in 11 cases, whereas in the remaining cases, Down syndrome was confirmed postnatally based on clinical assessment and karyotype analysis.

As the primary objective of this study was to evaluate the diagnostic performance of first-trimester prenatal screening parameters, an analytical sample was selected from the initial study population. Only cases with complete first-trimester screening data were included. The required variables were maternal age, nuchal translucency (NT), pregnancy-associated plasma protein A (PAPP-A), free β-human chorionic gonadotropin (free β-hCG), and ultrasound assessment of the fetal nasal bone.

Cases were excluded from the analytical sample if they had not undergone first-trimester screening or if the medical records contained incomplete or missing information on the required first-trimester ultrasound or biochemical screening parameters, including missing NT measurements, incomplete assessment of the fetal nasal bone, or unavailable biochemical screening results.

Following the application of these criteria, 53 cases were selected from the 80 live-born cases, and 18 cases were selected from the 23 prenatally diagnosed cases with subsequent pregnancy termination. Thus, the main analytical group comprised 71 pregnancies affected by Down syndrome, for which complete first-trimester clinical, biochemical, and ultrasound screening data were available.

For comparative analysis, 72 pregnancy cases were randomly selected from the medical records of pregnant women registered at the same regional and district perinatal centers during the study period. Complete first-trimester clinical, biochemical, and ultrasound screening data were also required for inclusion in the comparison group. The comparison group was selected randomly for analytical purposes, without stratification or selection based on the presence or absence of fetal congenital or chromosomal abnormalities.

During the initial retrospective review of medical records, cases of Patau syndrome (trisomy 13) and Edwards syndrome (trisomy 18) were also identified. However, these cases were not included in the final analytical sample because the primary objective of the study was to develop predictive models specifically for trisomy 21 (Down syndrome).

Thus, the final analytical sample comprised 143 cases: 71 pregnancies affected by Down syndrome and 72 randomly selected pregnancy cases included for comparative purposes. The analytical sample was constructed to evaluate the predictive and discriminative performance of first-trimester prenatal screening markers and to develop predictive models for Down syndrome rather than to estimate the population prevalence of Down syndrome in the region.

### 2.2. Clinical Data Collection

Clinical and prenatal screening data from the first trimester were retrospectively collected from the medical records of the pregnant women included in the study. The following parameters were analyzed: maternal age, nuchal translucency (NT) thickness, biochemical markers including pregnancy-associated plasma protein A (PAPP-A) and free β-human chorionic gonadotropin (free β-hCG), and fetal nasal bone hypoplasia or aplasia as an ultrasound marker.

The analyzed parameters were recorded as follows: maternal age in years; NT thickness in millimeters (mm); and PAPP-A and free β-hCG values as multiples of the median (MoM). Fetal nasal bone status was assessed during ultrasound examination as either normal or showing hypoplasia/aplasia.

Serum levels of PAPP-A and free β-hCG were measured using the Brahms Kryptor automated biochemical analyzer. The results were expressed as multiples of the median (MoM).

First-trimester ultrasound examinations were performed between 11 + 0 and 13 + 6 weeks of gestation using a Voluson E10 (GE Healthcare) expert-class ultrasound system. The ultrasound data included in the study were obtained from examinations performed by highly specialized specialists certified by the Fetal Medicine Foundation (FMF). Ultrasound assessment included measurement of NT thickness and evaluation of fetal nasal bone status.

### 2.3. Statistical Analysis

Continuous variables are presented as medians and interquartile ranges (IQRs), whereas categorical variables are described as absolute frequencies and percentages.

Multivariable logistic regression analysis was performed to assess the independent associations of first-trimester prenatal screening parameters with Down syndrome. Predictors were selected a priori based on their established clinical relevance in first-trimester screening for trisomy 21. Maternal age, NT, PAPP-A, and free β-hCG were entered into the regression models as continuous variables, whereas fetal nasal bone status was entered as a binary variable, with normal nasal bone status coded as 0 and hypoplasia or aplasia coded as 1. The results are presented as odds ratios (ORs) with 95% confidence intervals (95% CIs). Statistical significance was set at *p* < 0.05.

The diagnostic performance of the predictive models was evaluated using receiver operating characteristic (ROC) curve analysis. The discriminatory ability of the models was assessed by calculating the area under the ROC curve (AUC). Sensitivity and specificity were also calculated.

Two predictive models were developed during the study:

1. The first model included maternal age, NT, PAPP-A, and free β-hCG;
2. The second model included the above variables together with fetal nasal bone hypoplasia or aplasia.

Multivariable logistic regression and ROC analysis were used to assess the individual and combined diagnostic performance of the investigated markers for Down syndrome.

Model fit and predictive accuracy were assessed using the Hosmer–Lemeshow test, Brier score, and calibration curve. Internal validation was performed using the bootstrap method with 1000 resamples to evaluate model stability and internal validity under repeated resampling.

Decision curve analysis (DCA) was performed as an exploratory analysis to examine the net benefit of the evaluated models across different threshold probabilities.

Missing data were handled using a complete-case approach. Only pregnancies with complete information on all variables required for the predictive analyses were included. Of the 103 identified Down syndrome cases, 71 had complete first-trimester screening data and were included in the final analytical sample, while 32 cases were excluded because of incomplete medical records or missing screening information. The final analytical sample of 143 pregnancies contained complete data for all predictors included in the multivariable models; therefore, no additional observations were excluded from the regression analyses because of missing predictor values. No imputation procedures were performed.

### 2.4. Ethical Approval

This study was approved by the Ethical Committee of Khoja Akhmet Yasawi University (protocol N 4/26, dated 8 May 2026).

## 3. Results

A total of 143 pregnancies were included in the final analytical sample, comprising 71 pregnancies affected by Down syndrome and 72 randomly selected pregnancy cases included for comparative analysis. The descriptive clinical and first-trimester screening characteristics of the analytical sample are presented in Table 1.

The median maternal age was 33 years (IQR, 27–39), and the median NT thickness was 1.5 mm (IQR, 1.3–1.9). The median PAPP-A and free β-hCG values were 0.82 MoM (IQR, 0.51–1.25) and 1.05 MoM (IQR, 0.63–1.62), respectively. Fetal nasal bone hypoplasia or aplasia was identified in 22 (15.4%) pregnancies.

### 3.1. Multivariable Logistic Regression Analysis

The results of the multivariable logistic regression analysis are presented in Table 2.

The results of the multivariable logistic regression analysis are presented in Table 2. Maternal age and NT thickness showed statistically significant independent associations with Down syndrome. Each additional year of maternal age was associated with higher odds of Down syndrome (OR = 1.16, 95% CI 1.08–1.24; *p* < 0.001). Similarly, an increase in NT thickness was associated with higher odds of Down syndrome (OR = 3.01, 95% CI 1.65–5.48; *p* = 0.001). In contrast, PAPP-A (OR = 0.95, 95% CI 0.60–1.52; *p* = 0.830) and free β-hCG (OR = 1.20, 95% CI 0.85–1.69; *p* = 0.300) were not statistically significant predictors in the adjusted model.

### 3.2. Diagnostic Performance of Model 1

**Figure 1:**
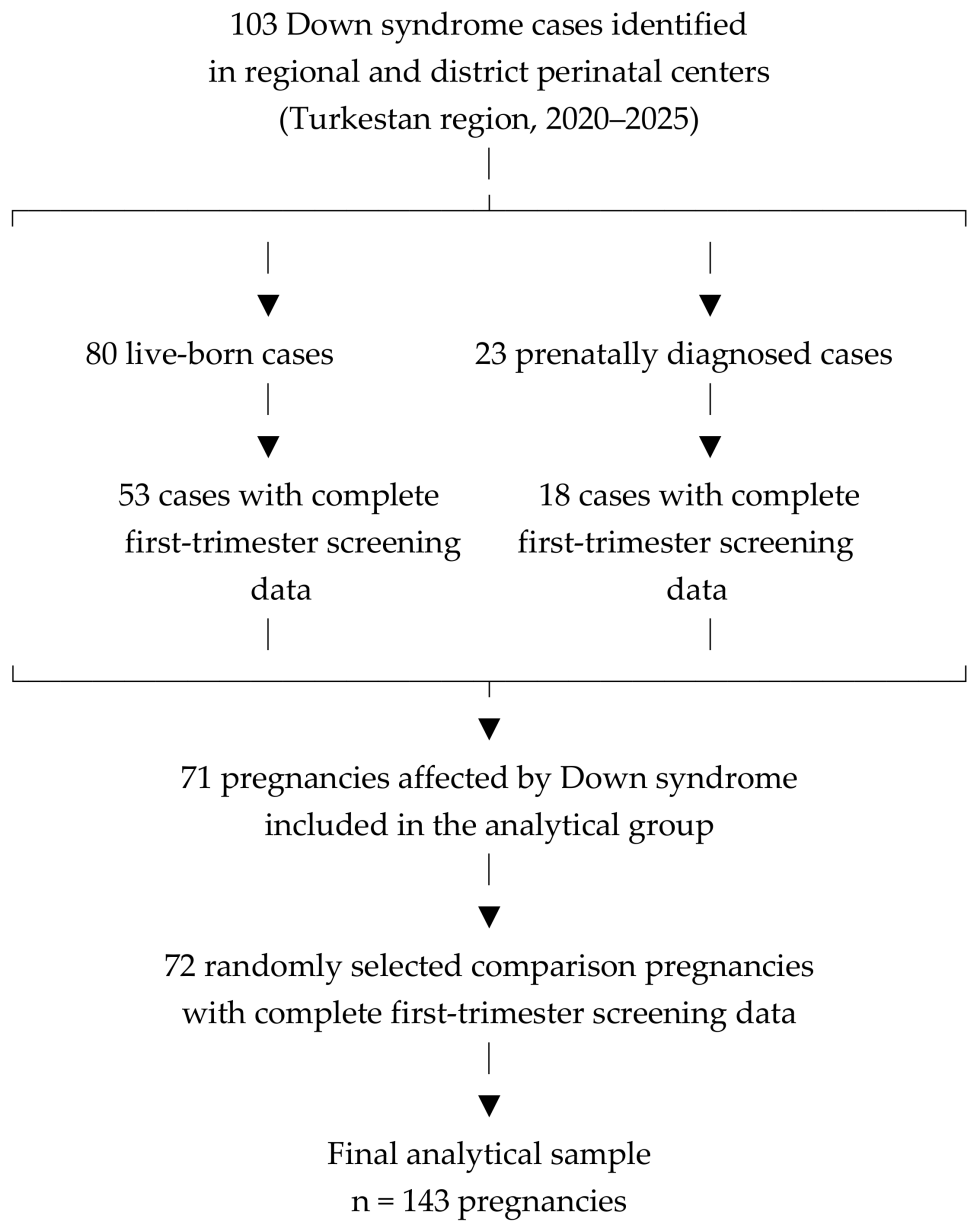
Flow diagram of participant selection and formation of the analytical sample. Excluded from the analytical sample (*n* = 32): • Incomplete medical records; • Missing first-trimester screening information.

The ROC analysis demonstrated good discrimination of the first exploratory model, which incorporated maternal age, NT thickness, PAPP-A, and free β-hCG (Table 3, Figure 1).

The ROC curve analysis demonstrated good discrimination of Model 1, which included maternal age, NT thickness, PAPP-A, and free β-hCG. The AUC was 0.855 (95% CI 0.793–0.917). At the reported cut-off value of 0.61, the sensitivity was 64.3%, and the specificity was 93.1%.

### 3.3. Diagnostic Performance of Model 2

The second model included maternal age, NT thickness, PAPP-A, free β-hCG, and fetal nasal bone hypoplasia/aplasia. The results of the multivariable logistic regression analysis for Model 2 are presented in Table 4.

**Figure 2:**
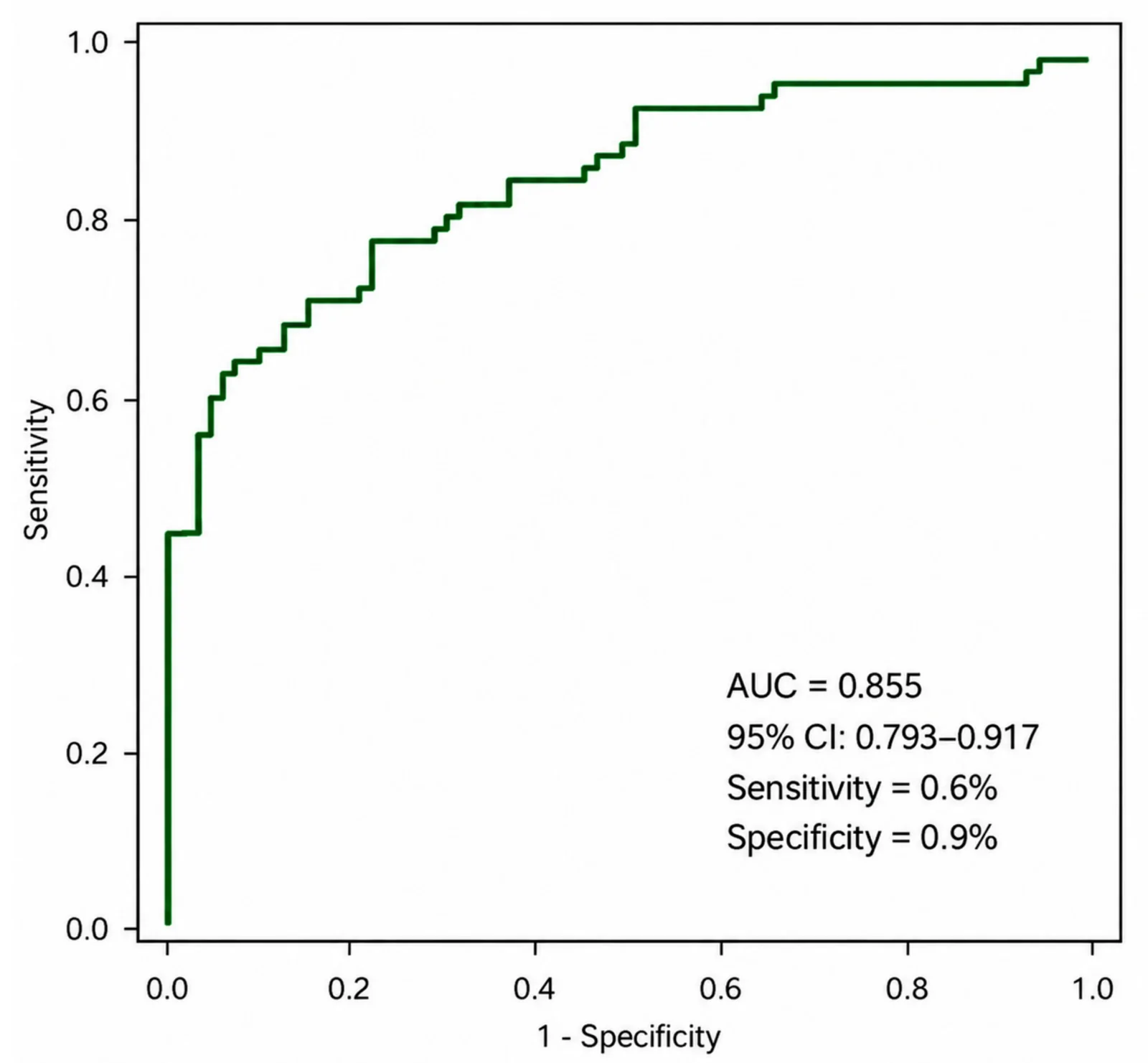
ROC curve of Model 1 for predicting Down syndrome.

Maternal age and NT thickness remained statistically significant predictors of Down syndrome. Maternal age was associated with higher odds of Down syndrome (OR = 1.20, 95% CI 1.12–1.31; *p* < 0.001), while increased NT thickness was also associated with higher odds of Down syndrome (OR = 5.79, 95% CI 2.23–19.20; *p* < 0.001). PAPP-A and free β-hCG were not statistically significant in the adjusted model. For nasal bone hypoplasia/aplasia, a stable OR and 95% CI could not be reliably estimated because of the rarity of the event and possible data separation. The ROC curve analysis of Model 2 demonstrated an AUC of 0.903 (95% CI 0.853–0.953), compared with 0.855 for Model 1. At the reported cut-off value, the sensitivity of Model 2 was 72.9%, and the specificity was 94.4% (Table 5, Figure 2).

Figure 2 shows the ROC curves of the two predictive models, with AUC values of 0.855 for Model 1 and 0.903 for Model 2.

### 3.4. Model Validation

The performance of Model 2 was further evaluated using calibration analysis and internal bootstrap validation. The Hosmer–Lemeshow goodness-of-fit test yielded a *p*-value of 0.985, indicating no statistically significant evidence of lack of fit. The Brier score was 0.12 (Table 6).

**Figure 3:**
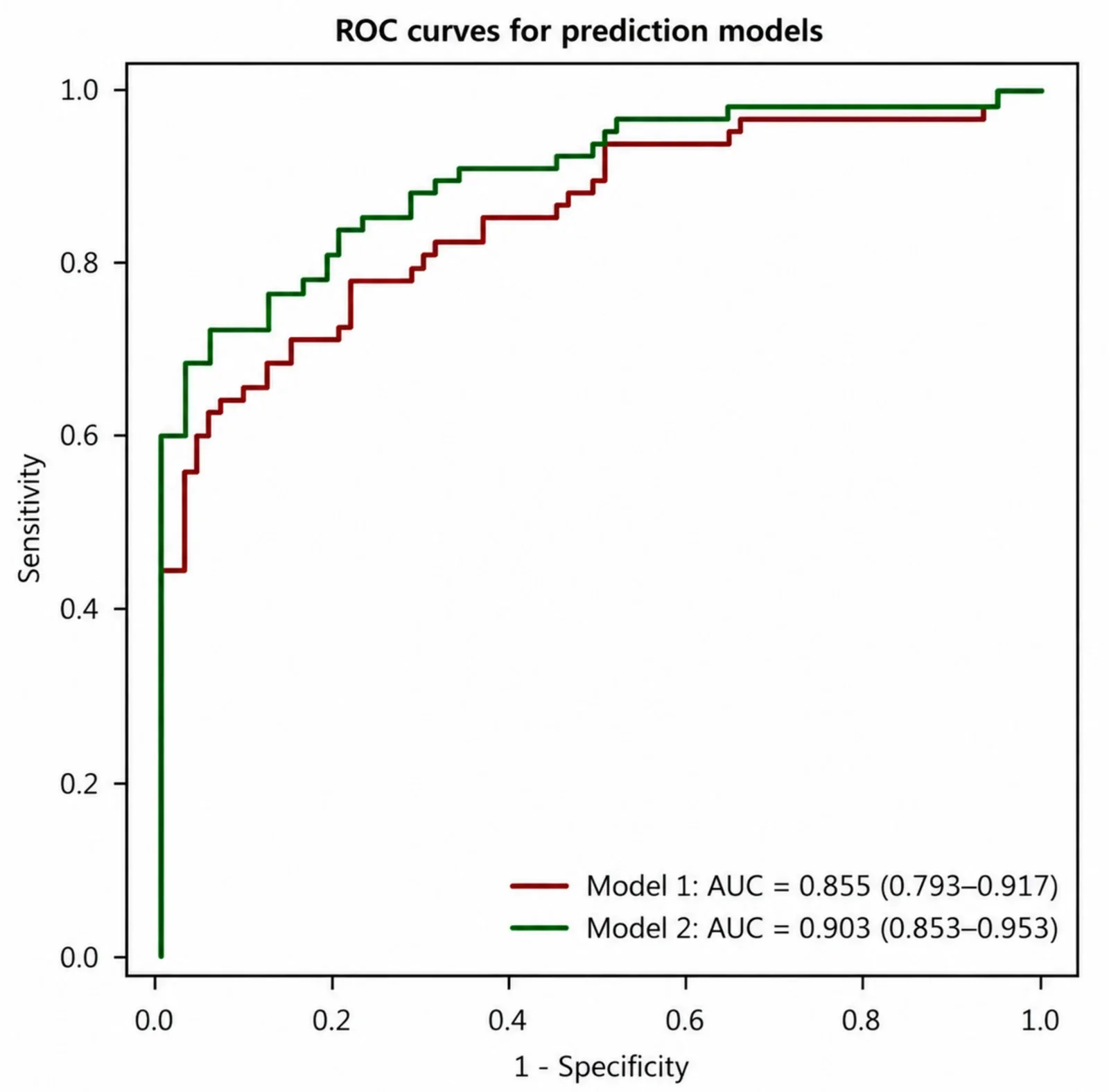
Comparison of the ROC curves of the two predictive models for Down syndrome.

Internal validation was performed using 1000 bootstrap resamples. After optimism correction, the AUC was 0.886. The optimism-corrected calibration slope was 0.856, and the calibration intercept was close to zero. These findings suggest that the model retained reasonable discriminatory performance after internal validation, although a calibration slope below 1 indicates some degree of optimism or overfitting in the model estimates. The calibration plot is presented in Figure 3.

### 3.5. Decision Curve Analysis

**Figure 4:**
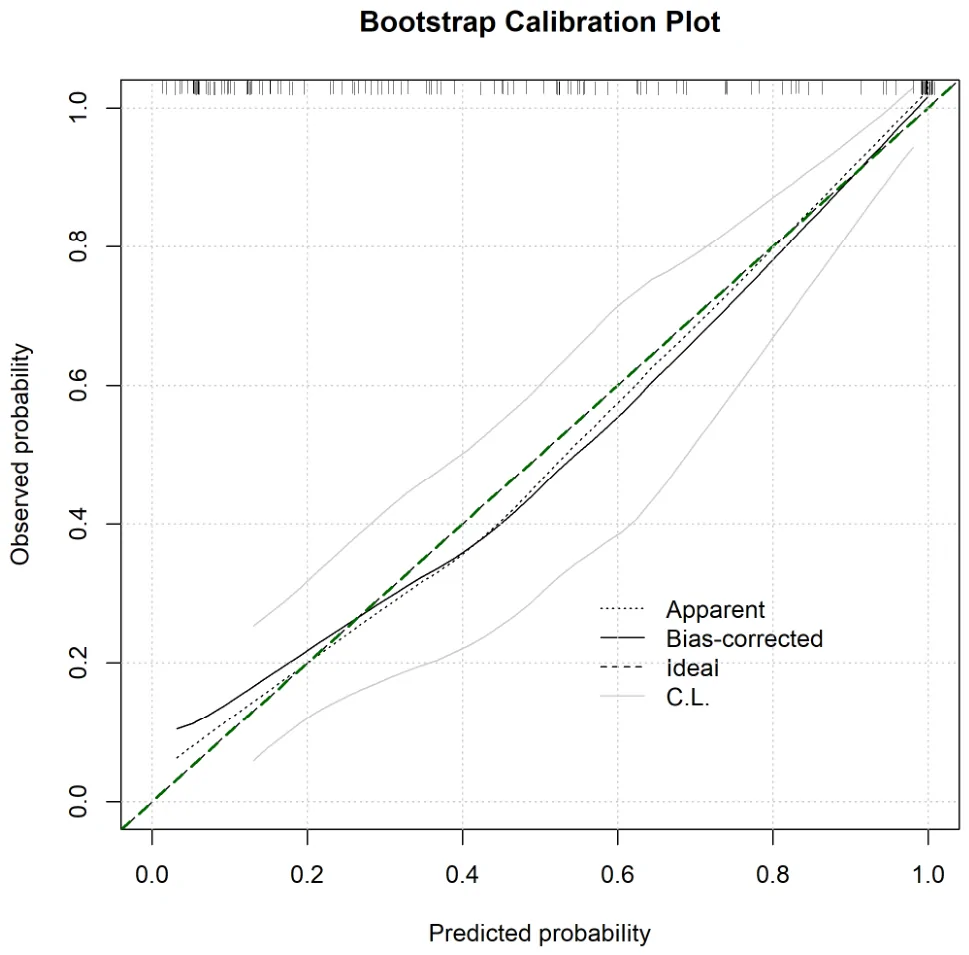
Calibration plot of Model 2 after internal bootstrap validation.

Decision curve analysis was performed to compare the net benefit of the two predictive models across a range of threshold probabilities (Figure 4). Within the analytical sample, Model 2 showed a higher estimated net benefit than Model 1 across the threshold probability range shown in the decision curve. However, because the analytical sample was constructed with an artificially high proportion of Down syndrome cases, these findings should not be directly interpreted as evidence of clinical utility in a routine prenatal screening population. 

**Figure 5:**
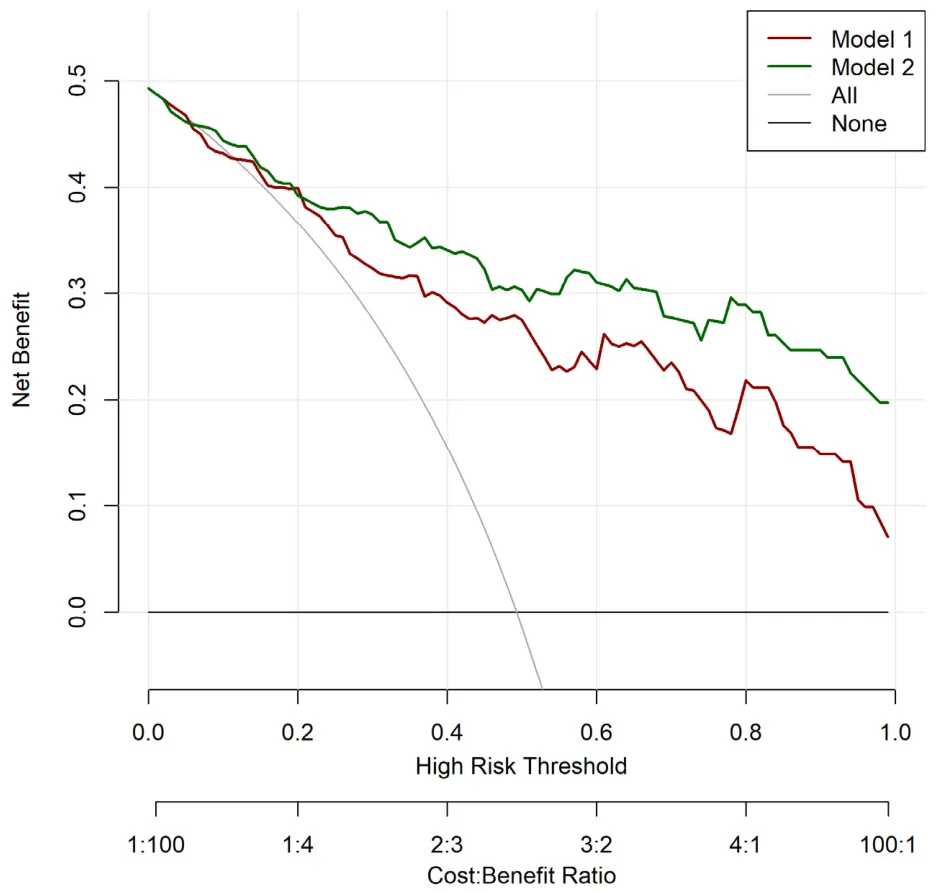
Decision curve analysis of the two predictive models for Down syndrome. Model 1 and Model 2 are shown as separate curves, while the “treat all” and “treat none” strategies are presented as reference strategies. The decision curves illustrate the estimated net benefit of each model across the range of threshold probabilities shown.

In Figure 5, Model 1 and Model 2 are represented by separate curves, while the “treat all” and “treat none” strategies are shown as reference strategies. The decision curves illustrate the estimated net benefit of each model across the indicated range of threshold probabilities.

## 4. Discussion

The obtained results indicate that maternal age and nuchal translucency were the main factors associated with Down syndrome in the present analytical sample. The identified association is consistent with current understanding of the pathogenesis of aneuploidies and is consistent with the results of large international studies, according to which increasing maternal age is associated with an increased risk of meiotic nondisjunction and, consequently, an increased incidence of trisomy 21 [16,17,18,19].

An equally important finding of the study was the association of NT with Down syndrome in the present analytical sample. Nuchal translucency thickening is recognized as one of the most informative ultrasound markers of the first trimester and is widely used in international prenatal screening programs. Our findings are consistent with the research of K. H. Nicolaides and the recommendations of The Fetal Society Medicine Foundation, according to which the combination of NT and maternal age significantly increases the effectiveness of early detection of Down syndrome [20,21].

Unlike ultrasound parameters, the biochemical markers PAPP-A and free β-hCG did not demonstrate independent statistical significance after adjustment for other factors in the present analytical sample. However, the absence of independent statistical significance in this study should not be interpreted as evidence of limited clinical value. These markers are used as components of combined prenatal screening strategies and contribute to risk assessment together with other maternal and screening parameters [22,23,24]. Therefore, biochemical markers should be considered as additional components of a comprehensive risk assessment.

Of particular interest is the comparison of the two predictive models. The inclusion of fetal nasal bone hypoplasia/aplasia was associated with a higher observed AUC for Model 2 (0.903) compared with Model 1 (0.855) [25,26]. However, this difference should be interpreted cautiously. A stable regression estimate for nasal bone hypoplasia/aplasia could not be obtained because of the limited number of pregnancies with this finding and possible data separation. Therefore, the observed increase in overall model discrimination should not be interpreted as evidence of an independent association of the nasal bone variable. Further evaluation in larger samples is required.

An important strength of the study is the comprehensive evaluation of model performance. In addition to ROC analysis, calibration assessment, internal bootstrap validation, and decision curve analysis (DCA) were performed. The results provided information on model performance within the present analytical sample. However, these findings should be interpreted cautiously, as the model was evaluated and internally validated within a relatively small retrospective sample with a non-representative proportion of Down syndrome cases. External validation in an independent and representative prenatal screening population is required before routine clinical application can be considered [27,28,29].

The practical significance of the study lies in its use of a regional sample from the Turkestan region, a high-birth-rate region of Kazakhstan. The findings may provide useful evidence for further evaluation of regional prenatal screening and risk assessment strategies. Prenatal screening services have become increasingly available in many countries, allowing for earlier and more accurate risk assessment before invasive diagnostic procedures are considered [30]. However, external validation in representative prenatal screening populations is required before the model can be considered for routine clinical application.

The limitations of this study should also be considered. First, the study’s retrospective design limits the ability to fully control for potential confounding factors. Second, the study was conducted in a single region and was based on a relatively small sample, necessitating caution when extrapolating the results to the entire population. No formal a priori sample size calculation was performed because the study was based on all eligible Down syndrome cases with complete first-trimester screening data identified during the retrospective study period. Nevertheless, the relatively small analytical sample may have limited the precision and stability of some regression estimates and may increase the risk of model overfitting. Therefore, further validation of the model in larger, independent populations is required. Future multicenter prospective studies involving a larger number of observations are needed, as well as a comparative evaluation of the developed model with modern noninvasive prenatal testing methods.

Beyond its diagnostic implications, the present study has important perspectives for the development of prenatal care services. The observed associations between maternal age, ultrasound markers, and Down syndrome may provide a basis for further research on regional prenatal risk assessment strategies. In regions with high birth rates, such as the Turkestan region, these findings may be relevant to the further refinement of prenatal risk assessment approaches. However, these potential applications require prospective external validation in representative prenatal screening populations and health-economic evaluation before widespread implementation in routine clinical practice.

Overall, the study results suggest that the highest diagnostic performance in the present analytical sample was achieved through a combined assessment of maternal age and ultrasound markers. The biochemical parameters did not demonstrate independent statistical significance in the multivariable analysis but remain established components of combined first-trimester screening strategies. This approach may contribute to improved Down syndrome risk stratification and further development of prenatal screening systems.

## 5. Conclusions

This study demonstrated that maternal age and fetal nuchal translucency (NT) thickness showed the strongest independent associations with Down syndrome in the present analytical sample.

The addition of nasal bone hypoplasia/aplasia to the evaluated model increased the AUC from 0.855 to 0.903 while maintaining high specificity. However, because the regression estimate for the nasal bone variable was unstable, this finding does not demonstrate an independent association between nasal bone hypoplasia/aplasia and Down syndrome.

The biochemical markers PAPP-A and free β-hCG did not demonstrate independent statistical significance after adjustment for maternal age and ultrasound findings in the present analytical sample. However, this should not be interpreted as evidence of limited clinical value, as these markers remain established components of combined first-trimester screening strategies.

The exploratory models demonstrated good discrimination and acceptable internal validation performance. However, external validation in independent and representative prenatal screening populations is required before routine clinical application can be considered.

These findings suggest that integrating maternal age with key ultrasound markers may improve prenatal risk stratification for Down syndrome within the present analytical sample.

## Figures and Tables

**Table 1:** Descriptive characteristics of the study sample (*n* = 143).

Variable	Meaning
Mother’s age, years	33 [27–39]
Nuchal translucency (NT) thickness, mm	1.5 [1.3–1.9]
PAPP-A (MoM)	0.82 [0.51–1.25]
Free β-hCG (MoM)	1.05 [0.63–1.62]
Hypoplasia/aplasia of the nasal bones, *n* (%)	22 (15.4%)
Down syndrome, *n*	71
Comparative group, *n*	72

Note: Continuous variables are presented as median and interquartile range (IQR), categorical variables as absolute number of observations and percentage (*n*, %).

**Table 2:** Multivariable logistic regression analysis for predictors of Down syndrome (Model 1).

Variable	OR	95% CI	*p*-Value
Mother’s age, years	1.16	1.08–1.24	<0.001
NT thickness, mm	3.01	1.65–5.48	0.001
PAPP-A (MoM)	0.95	0.60–1.52	0.83
Free β-hCG (MoM)	1.20	0.85–1.69	0.30

Note. OR is the odds ratio; 95% CI is the 95% confidence interval. OR values > 1 indicate an increased risk of Down syndrome, while OR < 1 indicates a decreased risk. Continuous variables (age, NT, biochemical markers) were analyzed as quantitative indicators.

**Table 3:** Diagnostic performance of Model 1 based on ROC curve analysis.

Indicator	Meaning
AUC (Area Under the Curve)	0.855
95% confidence interval (CI)	0.793–0.917
Cut-off	0.61
Sensitivity %	64.3
Specificity %	93.1

**Table 4:** Multivariable logistic regression analysis for predicting Down syndrome (Model 2).

Predictor	OR	95% CI	*p*
Mother’s age, years	1.20	1.12–1.31	<0.001
Increase in NT, mm	5.79	2.23–19.20	<0.001
PAPP-A (MoM)	0.98	0.49–1.94	0.953
Free β-hCG (MoM)	1.37	0.88–2.26	0.177
Hypoplasia/aplasia of the nasal bones	—	—	0.991

Abbreviations: OR, odds ratio; CI, confidence interval; NT, nuchal translucency; PAPP-A, pregnancy-associated plasma protein A; β-hCG, beta-human chorionic gonadotropin; MoM, multiples of the median. Note: Continuous predictors (maternal age, NT thickness, PAPP-A, and free β-hCG) were entered into the model as continuous variables. Nasal bone hypoplasia/aplasia was entered as a binary variable (0 = normal nasal bone; 1 = hypoplasia/aplasia). A stable OR and 95% CI could not be reliably estimated for nasal bone hypoplasia/aplasia because of the rarity of the event and possible data separation.

**Table 5:** Comparison of the diagnostic performance of the two predictive models for Down syndrome.

Model	Predictors	AUC (95% CI)	Cut-Off	Sensitivity, %	Specificity, %
Model 1	Age mothers + NT + PAPP-A + β-hCG	0.855 (0.793–0.917)	0.61	64.3	93.1
Model 2	Age mothers + NT + PAPP-A + β-hCG + hypoplasia/aplasia bones	0.903 (0.853–0.953)	0.58	72.9	94.4

**Table 6:** Performance and calibration measures of the predictive model for Down syndrome.

Indicator	Result
AUC	0.903
Hosmer–Lemeshow	*p* = 0.985
Brier score	0.12

## Data Availability

The data presented in this study are available on request from the corresponding authors. The data are not publicly available due to ethical restrictions.

## References

[1] World Health Organization (2023). Congenital Disorders. https://www.who.int/health-topics/congenital-anomalies.

[2] Pal A., Daly S.F. (2026). Down Syndrome. StatPearls [Internet].

[3] National Institute of Child Health and Human Development (2024). About Down Syndrome. https://www.nichd.nih.gov/health/topics/factsheets/downsyndrome.

[4] Hui L., Ellis K., Mayen D., Pertile M.D., Reimers R., Sun L., Vermeesch J., Vora N.L., Chitty L.S. (2023). Position statement from the International Society for Prenatal Diagnosis on the use of non-invasive prenatal testing for the detection of fetal chromosomal conditions in singleton pregnancies. Prenat. Diagn..

[5] American College of Obstetricians and Gynecologists’ Committee on Practice Bulletins—Obstetrics, Committee on Genetics, Society for Maternal-Fetal Medicine (2020). Screening for Fetal Chromosomal Abnormalities: ACOG Practice Bulletin, Number 226. Obstet. Gynecol..

[6] Nicolaides K.H. (2005). First-trimester screening for chromosomal abnormalities. Semin. Perinatol..

[7] Audibert F., Wou K., Okun N., De Bie I., Wilson R.D. (2024). Guideline No. 456: Prenatal Screening for Fetal Chromosomal Anomalies. J. Obstet. Gynaecol. Can..

[8] Ghaffari S.R., Tahmasebpour A.R., Jamal A., Hantoushzadeh S., Eslamian L., Marsoosi V., Fattahi F., Rajaei M., Niroomanesh S., Borna S. (2012). First-trimester screening for chromosomal abnormalities by integrated application of nuchal translucency, nasal bone, tricuspid regurgitation and ductus venosus flow combined with maternal serum free β-hCG and PAPP-A: A 5-year prospective study. Ultrasound Obstet. Gynecol..

[9] National Center for Biotechnology Information (2025). Prenatal Genetic Screening. StatPearls [Internet].

[10] Bureau of National Statistics of the Republic of Kazakhstan (2025). Administrative-Territorial Units of the Republic of Kazakhstan. https://stat.gov.kz/upload/iblock/89b/3khm5i91ypm6zpg45vvbknmq43tnwgrm/%D0%92-18-14-%D0%93(eng).pdf.

[11] Amiel A., Tarabeih M. (2023). Prenatal Tests Undertaken by Muslim Women Who Underwent IVF Treatment, Secular Versus Religious: An Israeli Study. J. Relig. Health.

[12] Amiel A., Tarabeih M. (2023). Prenatal Testing and Pregnancy Termination Among Muslim Women Living in Israel Who Have Given Birth to a Child with a Genetic Disease. J. Relig. Health.

[13] Amiel A., Na’amnih W., Tarabeih M. (2023). Prenatal Diagnosis and Pregnancy Termination in Jewish and Muslim Women with a Deaf Child in Israel. Children.

[14] Sun Y., Zhang L., Dong D., Li X., Wang J., Yin C., Poon L.C., Tian J., Wu Q. (2021). Application of an individualized nomogram in first-trimester screening for trisomy 21. Ultrasound Obstet. Gynecol..

[15] Ayazbekov A., Oshibayeva A., Ozkan S., Taskinova G., Taubekova M. (2026). Registered Prevalence and Regional Characteristics of Congenital Anomalies in the Turkistan Region. Int. J. Environ. Res. Public Health.

[16] Morris J.K., Mutton D.E., Alberman E. (2002). Revised estimates of the maternal age specific live birth prevalence of Down’s syndrome. J. Med. Screen..

[17] Elmerdahl Frederiksen L., Ølgaard S.M., Roos L., Petersen O.B., Rode L., Hartwig T., Ekelund C.K., Vogel I., Danish Central Cytogenetics Registry Study Group (2024). Maternal age and the risk of fetal aneuploidy: A nationwide cohort study of more than 500 000 singleton pregnancies in Denmark from 2008 to 2017. Acta Obstet. Gynecol. Scand..

[18] Loane M., Morris J.K., Addor M.C., Arriola L., Budd J., Doray B., Garne E., Gatt M., Haeusler M., Khoshnood B. (2013). Twenty-year trends in the prevalence of Down syndrome and other trisomies in Europe: Impact of maternal age and prenatal screening. Eur. J. Hum. Genet..

[19] Wang F.F., Li Y., Liu M.M., Han Y.M., Sun Z.W., Wang C., Wang L.L., Guo M., Li P.L. (2024). Chromosomal analysis in pregnant women of advanced maternal age: Indications for prenatal diagnosis. J. Physiol. Pharmacol..

[20] Nicolaides K.H. (2011). Screening for fetal aneuploidies at 11 to 13 weeks. Prenat. Diagn..

[21] The Fetal Medicine Foundation Screening for Chromosomal Abnormalities. https://fetalmedicine.org.

[22] Benn P., Cuckle H., Pergament E. (2013). Non-invasive prenatal testing for aneuploidy: Current status and future prospects. Ultrasound Obstet. Gynecol..

[23] Carbone L., Cariati F., Sarno L., Conforti A., Bagnulo F., Strina I., Pastore L., Maruotti G.M., Alviggi C. (2021). Non-Invasive Prenatal Testing: Current Perspectives and Future Challenges. Genes.

[24] Wald N.J., Bestwick J.P. (2013). Incorporating DNA sequencing into current prenatal screening practice for Down’s syndrome. PLoS ONE.

[25] Cicero S., Curcio P., Papageorghiou A., Sonek J., Nicolaides K.H. (2001). Absence of nasal bone in fetuses with trisomy 21 at 11–14 weeks of gestation: An observational study. Lancet.

[26] Kagan K.O., Hoopmann M., Sonek J. (2025). Second trimester soft markers: Still worth to be mentioned?. Arch. Gynecol. Obstet..

[27] Van Calster B., Wynants L., Verbeek J.F.M., Verbakel J.Y., Christodoulou E., Vickers A.J., Roobol M.J., Steyerberg E.W. (2018). Reporting and Interpreting Decision Curve Analysis: A Guide for Investigators. Eur. Urol..

[28] Van Calster B., McLernon D.J., van Smeden M., Wynants L., Steyerberg E.W., Topic Group ‘Evaluating diagnostic tests and prediction models’ of the STRATOS initiative (2019). Calibration: The Achilles heel of predictive analytics. BMC Med..

[29] Heus P., Reitsma J.B., Collins G.S., Damen J.A.A.G., Scholten R.J.P.M., Altman D.G., Moons K.G.M., Hooft L. (2020). Transparent Reporting of Multivariable Prediction Models in Journal and Conference Abstracts: TRIPOD for Abstracts. Ann. Intern. Med..

[30] Wilmot H.C., de Graaf G., van Casteren P., Buckley F., Skotko B.G. (2023). Down syndrome screening and diagnosis practices in Europe, United States, Australia, and New Zealand from 1990–2021. Eur. J. Hum. Genet..

